# Jump-Squat and Half-Squat Exercises: Selective Influences on Speed-Power Performance of Elite Rugby Sevens Players

**DOI:** 10.1371/journal.pone.0170627

**Published:** 2017-01-23

**Authors:** Irineu Loturco, Lucas A. Pereira, José E. Moraes, Katia Kitamura, César C. Cal Abad, Ronaldo Kobal, Fábio Y. Nakamura

**Affiliations:** 1 NAR—Nucleus of High Performance in Sport, São Paulo, SP, Brazil; 2 CBRu—Brazilian Rugby Confederation; São Paulo, SP, Brazil; 3 State University of Londrina, Londrina, PR, Brazil; Universita degli Studi di Verona, ITALY

## Abstract

The aim of this study was to evaluate the relation between the maximum mean propulsive power (MPP) obtained in the loaded jump squat (JS) and half squat (HS) exercises and functional performances in vertical jumps, 40 m linear speed (VEL) and change-of-direction (COD) tests, using the median split technique. Twenty-two male rugby sevens players from the Brazilian National Olympic Team (Rio-2016) performed vertical jumping tests (squat and countermovement jumps [SJ and CMJ]), JS and HS exercises, COD speed test and sprinting velocity in 40 m, in this order. Based on the results of the MPP in the JS and HS exercises the participants were divided, using the median split, into four groups as follows: higher JS, lower JS, higher HS, and lower HS. Between-group differences in the functional tasks were detected via magnitude-based inferences. The athletes with higher MPP in the JS were capable of jumping higher and sprinting faster (including the COD speed test) than their weaker counterparts. This pattern was not observed in the HS exercise. To conclude, JS was shown to be more connected to sprinting, COD speed and jumping abilities than HS in elite rugby sevens players and should be preferred for assessing and possibly training elite athletes needing to improve speed-power related abilities.

## Introduction

Specific performance in top-level sports depends on adequate training methods and precise training load adjustments [1]. In this regard, several studies have been conducted to determine the impact of distinct training strategies and loading patterns on the neuromechanical capacities of elite athletes [2–4]. Additionally, some research has already pointed out that some types of resistance exercises (i.e., ballistic or non-ballistic movements) may present different effects and relationships with speed-power related performance [5]. Whereas jump exercises are regularly chosen because of their similarity to sport-specific movements and relatively low complexity [6], the half squat might be recommended due to its inherent capacity to improve both “typical force-velocity relationships” [7] and, thus, athletic performance (e.g., linear speed and COD ability). This issue is critical to performance in team sports [8] since the requirements of speed and turns (i.e., change of direction) have been steadily increasing in competitions [9,10], and these capacities are thought to determine match outcomes [11]. Accordingly, determining the most effective exercises and loads that lead to enhancements in sport-specific force-velocity qualities has become a relevant topic in the scientific literature [12].

For instance, a recent study exploring the particular effects of loaded jump squats and half squats in elite soccer players revealed that both exercises are capable of reducing the decrements in speed-power capacities that commonly occur throughout professional soccer preseasons[13]. Also, Hori et al. [14] demonstrated that standard weighted jump squats may cause larger adaptations in the force applied at high velocities (peak torque during isokinetic knee flexion at 300°^.^s^-1^), suggesting that this exercise can be used specifically to provoke positive changes in the low-force/high-velocity portion of the force-velocity curve. Similarly, Nibali et al. [12] proposed that athletes who are required to produce high velocities against high external loads (e.g., rugby players overcoming an opposing defenders tackle) be tested and trained at heavier loads, more specifically using loaded jump squats. On the other hand, training using half squats with heavy loads (80–90% of 1 repetition maximum) has also been shown to enhance jump and sprint performance in soccer players [7]. Therefore, it is currently unclear which exercise mode (ballistic vs. non-ballistic) is better suited to improve field-based performance indicators in team sports players.

Another common way to examine the applicability of a given exercise in sport science is by assessing its relations with specific athletic capacities [15]. For instance, it has been reported that loaded jump squats are closely associated with speed tests and actual sprint performance in top-level sprinters [16,17]. In a classic correlational study, Wisløff et al. [18] showed that maximal strength in half squats is strongly associated with sprinting speed and jumping height in high-level soccer players. Nevertheless, in spite of the evident importance of correlational investigations, some authors have been cautious regarding the interpretation of these outcomes, since correlations do not necessarily imply cause and effect [19,20].

An alternative and applied strategy for estimating the influence of a particular exercise on actual sport performance is analyzing the data provided by the median split technique [21,22]. By using this calculation, researchers can suitably group the athletes according to their physical capacities, defining the “lower and upper bounds of performances” in a series of functional tests. Under this perspective, it seems reasonable to consider that higher levels of performance in two or more measurements should be directly interconnected, presenting reciprocal and consistent relations between them. Hence, this study aimed to evaluate (and classify) via median split calculation the performance of top-level rugby sevens players in a series of neuromechanical tests (i.e., half squat, loaded jump squat, vertical jump, linear speed and change-of-direction tests). After considering “loaded jump squats” and “half squats” as fixed factors and separating the subjects according to their higher or lower levels of performance in these exercises, we tried to detect the presence of analogous patterns between their respective outputs (i.e., mean propulsive power) and field-based speed and jump assessments. Considering the effectiveness of ballistic exercises (i.e., loaded jump squats) in enhancing performance [23] and their strong correlations with speed-power related capacities [16,18], we hypothesized that higher performances in loaded jump squats would necessarily imply in higher performances in sprinting (except for COD speed) and jumping abilities in highly selected rugby sevens players, whose match-related performance is highly dependent on speed and power qualities.

## Materials and Methods

### Participants

Twenty-two male rugby sevens players (25.6 ± 3.9 years; 87.5 ± 7.6 kg; 179.5 ± 6.7 cm) from the Brazilian National Olympic Team (Rio-2016) took part in this study. The rugby players were tested during the competitive phase, between the stages of the Rugby Sevens World Series, suggesting that the athletes were close to peak performance. The athletes were briefed about the procedures of this study before signing an informed consent form. This study was performed in accordance with the ethical standards of the Helsinki Declaration and was approved by the Anhanguera-Bandeirante University Ethics Committee.

### Study design

In this cross-sectional study, all athletes were previously familiarized with the experimental procedures and involved in the same training routine in the weeks prior to the study. The athletes arrived at the sports laboratory in a fasting state for 2 h and free of caffeine or alcohol consumption for at least 24 h. The assessments were performed in the following order: vertical jumping tests (squat and countermovement jumps [SJ and CMJ]); maximum MPP in the loaded jump squat (JS) and half squat (HS) exercises; change of direction (COD) speed test and sprinting velocity (VEL) in 40 m. An interval of at least 30 min was allowed between exercise tests. Before performing the test battery, the athletes completed a 20-min general standardized warm-up, including 15-min of general exercises (i.e., 10-min running at a moderate pace (between 9–10 km.h^-1^) followed by 5-min of lower-limb active stretching) and, prior to each test, 5-min of test-specific exercises (e.g., submaximal JS and HS attempts prior to the JS and HS exercise tests).

### Vertical jumping tests

Vertical jumping height was determined using both SJ and CMJ. In the SJ, subjects were required to remain in a static position with a 90° knee flexion angle for 2s before jumping. In the CMJ, the rugby players were instructed to execute a downward movement followed by a complete extension of the legs. The SJ and CMJ were executed with the hands fixed on the hips. All jumps were performed on a contact platform (Smart Jump; Fusion Sport, Coopers Plains, Australia) with the obtained flight time (t) being used to estimate the height of the rise of the body’s center of gravity (h) during the vertical jump (i.e., h = gt^2^/8, where g = 9.81 m∙s^-2^). A total of five attempts were allowed for each jump, interspersed with a15s interval. The best attempts at SJ and CMJ were retained.

### Mean propulsive power

Mean propulsive power (MPP) was measured in the JS and HS exercises; all performed on a Smith-Machine (Hammer Strength Equipment, Rosemont, IL, USA). The athletes were instructed to execute three repetitions at maximal velocity for each load, with ≈ 5min interval provided between sets. The test started at a load corresponding to 40% of the individual body mass. A load of 10% of body mass was gradually added in each set until a clear decrement in the MPP was observed. In the JS, the athletes executed a knee flexion until the thigh was parallel to the ground and, after a verbal command, jumped as fast as possible without their shoulder losing contact with the barbell. The HS was executed in a similar fashion to the JS, except that the subjects were instructed to move the bar as fast as possible without losing foot contact with the ground. To determine MPP, a linear transducer (T-Force, Dynamic Measurement System; Ergotech Consulting S.L., Murcia, Spain) was attached to the Smith-Machine bar. The maximum MPP output attained in each exercise was considered for further analysis. The technical specification of the MPP analysis, its calculation, and the respective validity of the equipment used to perform this measurement have been previously reported in the literature [13,24,25]. The MPP divided by the athletes’ body mass (MPP REL) obtained in each exercise were used for analysis purposes.

### Sprinting velocity

Five pairs of photocells (Smart Speed, Fusion Equipment, AUS) were positioned at distances of 0, 5, 10, 30, and 40 m along the sprinting course. The rugby players sprinted twice, starting from a standing position 0.3 m behind the starting line. To avoid weather influences, the sprint tests were performed on an indoor running track. A 5-min rest interval was allowed between the two attempts and the fastest time was considered for the analyses.

### Zig-zag change of direction (COD) speed test

**Fig 1:**
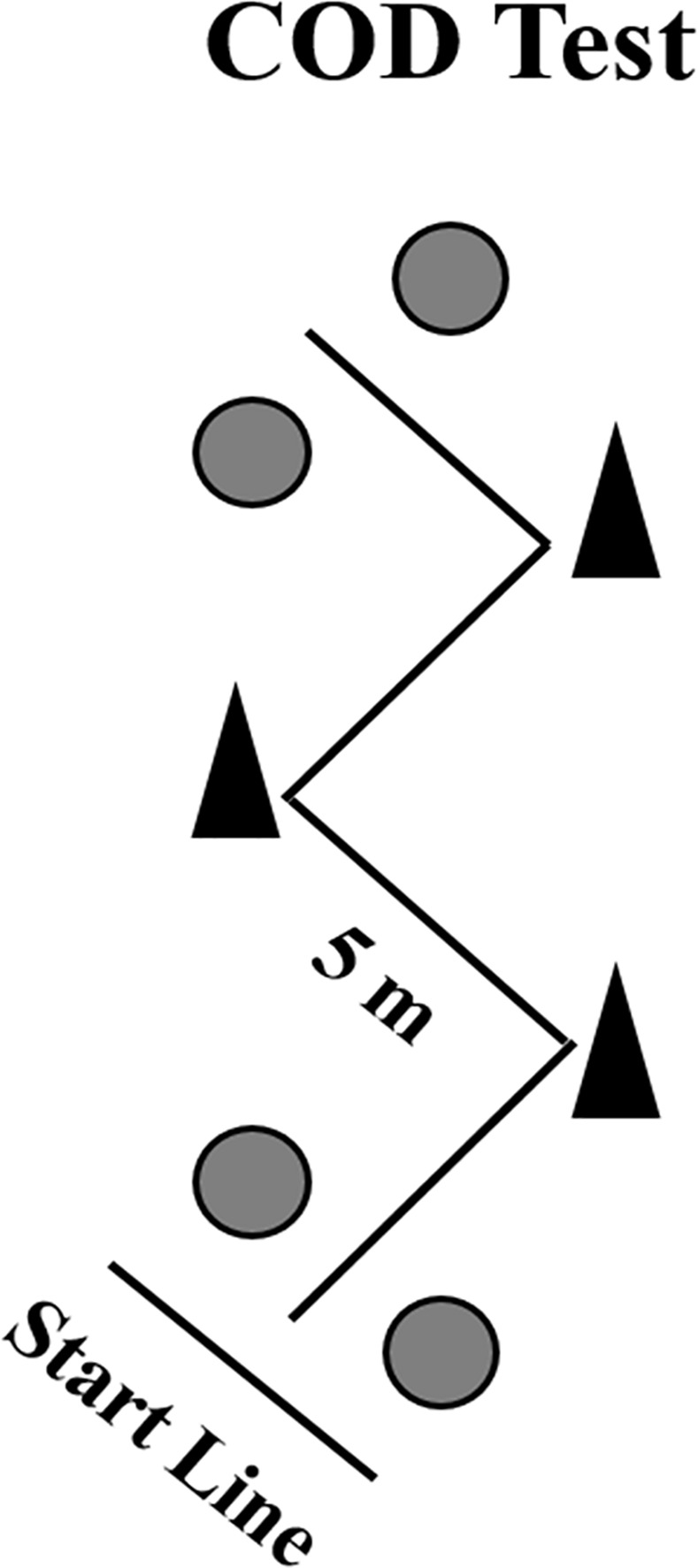
A schematic presentation of the Zig-zag COD speed test. The grey circles represent the position of the photocells.

The COD course consisted of four 5 m sections marked with cones set at 100° angles, on an indoor court (Fig 1). The athletes were required to decelerate and accelerate as fast as possible without losing body stability. Two maximal attempts were performed with a 5-min rest interval between attempts. Starting from a standing position with the front foot placed 0.3 m behind the first pair of photocells (i.e., starting line), the athletes ran and changed direction as quickly as possible, until crossing the second pair of photocells, placed 20 m from the starting line [26]. The fastest time from the two attempts was retained for analyses.

### Statistical analysis

Data are presented as means ± standard deviation. Based on the results of the MPP REL in the JS and HS exercises the participants were divided, using median split analysis, into four groups as follows: higher JS, lower JS, higher HS, and lower HS. The differences in the variables tested (performances in vertical jumps, 40-m sprint and COD tests), comparing higher and lower JS groups and higher and lower HS groups were analyzed using the magnitude based-inference method [27]. The smallest worthwhile change (SWC) was obtained by multiplying the between athlete SD x 0.2 [27] for each variable tested. The quantitative chances of finding differences in the variables tested, using the outcomes in raw units, were assessed qualitatively as follows: <1%, almost certainly not; 1% to 5%, very unlikely; 5% to 25%, unlikely; 25% to 75%, possible; 75% to 95%, likely; 95% to 99%, very likely; >99%, almost certain. If the chances of having better and poorer results were both >5%, the true difference was assessed as unclear. The magnitudes of the differences in the variables tested were expressed as standardized mean differences (Cohen’s *d*) and their respective confidence intervals (CI)[28]. Threshold values for Cohen’s *d* statistics were: <0.25, 0.25–0.50, 0.50–1, and >1 for trivial, small, moderate, and large, respectively[29].

## Results

The optimal loads achieved in each exercise were 75.0 ± 11.6 kg and 81.5 ± 19.8 kg, for the JS and HS, respectively. The higher JS group demonstrated an *almost certainly* higher MPP REL in the JS exercise than the lower JS group (12.3 ± 1.0 W^.^kg^-1^ vs. 10.0 ± 1.1 W^.^kg^-1^; SWC: ± 0.22; mean difference [MD, 90% CI]: 2.30 [1.53; 3.08]; ES [90% CI]: 1.95 [1.30; 2.61]). Similarly, the higher HS group demonstrated an *almost certainly* higher MPP REL in the HS exercise than the lower HS group (11.0 ± 1.3 W^.^kg^-1^ vs. 8.3 ± 1.0 W^.^kg^-1^; SWC: ± 0.19; MD [90% CI]: 2.69 [1.83; 3.54]; ES [90% CI]: 2.58 [1.76; 3.40]).

**Fig 2:**
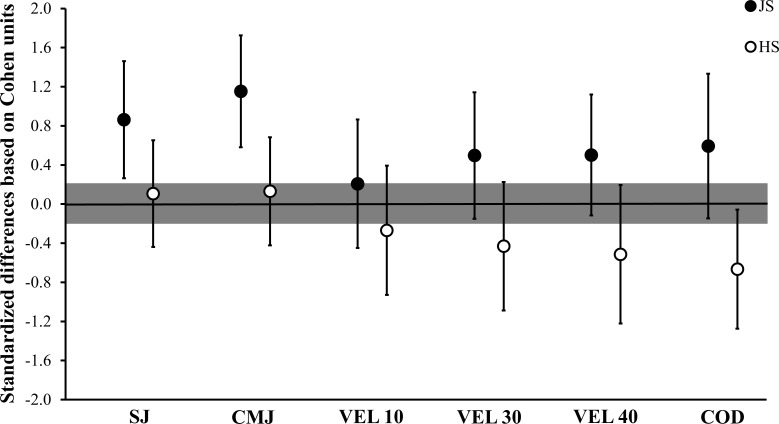
Standardized differences of the squat and countermovement jumps (SJ and CMJ, respectively), sprinting velocity (VEL) in 10, 30, and 40 m, and change of direction (COD) speed test between the higher and lower loaded jump squat (JS) groups and between the higher and lower half-squat (HS) groups. The grey area represents the smallest worthwhile change (0.20) based on Cohen’s principles.

Table 1 demonstrates the comparisons of the SJ, CMJ, sprinting velocities in 10, 30 and 40 m, and COD speed test between the higher and lower JS groups, as well as between the higher and lower HS groups. The performance in the SJ was *very likely* greater in the higher JS group than in the lower JS group (SWC: ± 0.90; MD [90% CI]: 4.22 [1.29; 7.14]), and the CMJ was *almost certainly* higher in the higher JS group in comparison to the lower JS group (SWC: ± 0.83; MD [90% CI]: 5.17 [2.60; 7.73]). The comparisons of the SJ and CMJ, between the higher HS and lower HS groups were all rated as *unclear* (SWC: ± 1.34; MD [90% CI]: 0.78 [-3.19; 4.74]; SWC: ± 1.25; MD [90% CI]: 0.88 [-2.85; 4.62], for SJ and CMJ, respectively). In addition, the comparisons of the sprinting velocity in 10 m between the higher and lower JS groups, and between the higher and lower HS groups were all rated as *unclear* (SWC: ± 0.04; MD [90% CI]: 0.04 [-0.09; 0.17]; SWC: ± 0.03; MD [90% CI]: -0.04 [-0.15; 0.07], for the comparisons between JS and HS groups, respectively). On the other hand, the sprinting velocities in 30 and 40 m and in the COD speed were *likely* higher in the higher JS group in comparison to the lower JS group (SWC: ± 0.04; MD [90% CI]: 0.11 [-0.03; 0.25]; SWC: ± 0.05; MD [90% CI]: 0.13 [-0.03; 0.29]; SWC: ± 0.03; MD [90% CI]: 0.11 [-0.03; 0.25], for velocity in 30 and 40 m, and COD speed, respectively). In contrast, the comparison of the VEL 30 m between the higher HS and lower HS groups was rated as *unclear* (SWC: ± 0.04; MD [90% CI]: -0.09 [-0.23; 0.05]). Finally, the higher HS group demonstrated *likely* lower VEL 40 m and COD speed than the lower HS group (SWC: ± 0.04; MD [90% CI]: -0.11 [-0.27; 0.04]; SWC: ± 0.03; MD [90% CI]: -0.10 [-0.19; -0.01], for velocity in 40 m and COD speed, respectively). Fig 2 depicts the standardized differences based on Cohen’s *d* units in the SJ, CMJ, VEL, 10, 30, and 40 m and the COD speed between the higher and lower JS and the higher and lower HS groups.

**Table 1:** Vertical jumps (SJ and CMJ), sprinting velocity (VEL) and change of direction (COD) test performances in the higher and lower loaded jump squat (JS) groups and in the higher and lower half-squat (HS) groups.

	Higher JS	Lower JS	% of chances higher/trivial/lower	Higher HS	Lower HS	% of chances higher/trivial/lower
SJ (cm)	45.8 ± 3.3	41.5 ± 4.5	96/04/00 *Very Likely*	43.7 ± 3.3	42.9 ± 6.7	38/45/17 *Unclear*
CMJ (cm)	48.6 ± 2.6	43.4 ± 4.1	100/00/00 *Almost Certainly*	45.6 ± 3.3	44.7 ± 6.2	41/43/16 *Unclear*
VEL 10 m (m^.^s^-1^)	5.97 ± 0.17	5.93 ± 0.18	51/34/15 *Unclear*	5.87 ± 0.14	5.92 ± 0.15	12/31/57 *Unclear*
VEL 30 m (m^.^s^-1^)	7.56 ± 0.18	7.45 ± 0.20	78/18/04 *Likely*	7.41 ± 0.18	7.50 ± 0.20	06/22/76 *Unclear*
VEL 40 m (m^.^s^-1^)	7.91 ± 0.20	7.78 ± 0.24	80/17/03 *Likely*	7.75 ± 0.22	7.86 ± 0.20	05/18/77 *Likely*
COD speed (m^.^s^-1^)	3.85 ± 0.20	3.74 ± 0.17	82/14/04 *Likely*	3.65 ± 0.09	3.75 ± 0.13	01/09/90 *Likely*

## Discussion

This is the first study to use median split analysis to search for reciprocal relations between the levels of performance in ballistic (i.e., loaded jump squats) and traditional (i.e., half squats) strength-power exercises and the levels of performance in specific speed and jump tests. The main finding reported herein is that there is a direct connection between JS and functional field-based assessments.

As expected, the athletes able to produce superior levels of muscle power in loaded jump squats were equally capable of jumping higher and sprinting faster than their weaker counterparts (i.e., lower JS group). Indeed, it seems that performance in this exercise is strictly connected with performance in motor tasks executed at high velocities, such as maximal sprints and unloaded vertical jumps. From a functional standpoint, it is reasonable to assume that the mechanical characteristics of the ballistic exercises (which do not present deceleration phases during their concentric portions) [24] are more related to sport-specific movements directly influenced by the rates of acceleration achieved throughout the complete ranges of motion [5], such as jumps and short sprints. It is important to emphasize that the traditional HS presents an inherent “braking phase” [24,25] during its upward portion, which may compromise its relation/connection with motor tasks executed at very-high velocities [5,30,31].

Curiously, higher performances in the HS were inversely related to higher performances in linear and COD speed. Although we do not consider that this occurrence implies causality or even a direct relationship, it reinforces the notion that this exercise has a limited role in promoting positive adaptations in athletic performance. In fact, in a classic study defining the best predictors of sports speed, Cronin and Hansen [32] indicated that mechanical measures collected from traditional squats cannot adequately express all the mechanisms responsible for performance in some specific functional tasks, such as maximal sprints. According to the authors, for significantly enhancing speed ability in elite athletes, improvement in “power to weight ratio” via loaded JS training should be prioritized. Similar results were found by Baker and Nance [33] and by Costill et al. [34], who failed to find significant correlations between HS measures and sprinting speed in professional rugby players and college football players. Importantly, all these correlational data have the same limitation as our median split approach, since cross-sectional analyses do not allow us to draw definitive conclusions about training efficacy. Nonetheless, the median split analysis can cluster players with distinct abilities, in occasions where (not necessarily) the independent and dependent variables are significantly correlated [22]. Its application is quite intuitive in sport settings, since technical staff could organize athletes in groups according to their individual qualities, delivering training strategies directly related to their respective physical and physiological traits.

The necessity to perform prospective investigations to confirm cross-sectional observations is a basic requirement in science. In this regard, several studies have confirmed the chronic effectiveness of JS on improving sports performance [23,35,36]. Remarkably, it seems that these positive effects are independent of the “JS loading condition/variation”, although this parameter may provoke specific/distinct adaptations in the force-velocity curve. For instance, Loturco et al. [23] have shown that increasing JS velocity (with the aid of elastic bands) favors adaptations in the high-velocity/low-force portion of the force-velocity curve, whereas reducing JS velocity (by using a traditional loading strategy) favors adaptations in the low-velocity/high-force portion of the curve. Equally, McBride et al. [35] have suggested that the movement velocity plays a determinant role in enhancing speed-related capacities, after comparing the effects of an 8-week training program with heavy- vs. light-load JS on numerous physical performance assessments. Therefore, it appears that not only the exercise selection but also the loading strategy adopted by the coaches may have a key function in delineating the specific neuromechanical adaptations induced by a given training scheme.

Notably, a recent study conducted with top-level soccer players has demonstrated that JS is superior to HS for reducing the decrements in speed which commonly occur following a preseason conditioning phase [13]. This assumption should be tested and revisited under other experimental designs (i.e., different training phases and/or longer interventions); however, unquestionably, this finding highlights the potential effectiveness of JS to increase athletic performance. As aforementioned, this superiority may be attributed to the kinematic/kinetic characteristic of this ballistic exercise, which circumvents any deceleration phase by requiring subjects to accelerate throughout the complete range of motion toward the takeoff [5,31]. These same mechanical particularities can be related to the greater jumping ability (for SJ and CMJ measures) presented by the higher JS group. Likewise, these data are in full accordance with the close relationships usually found between sprinting speed and vertical jumping performances [16,17].

Unexpectedly, higher performances in the JS were also related to higher COD speed performances. In spite of the multifactorial nature of this ability [37,38], it seems that rugby players with greater levels of relative power in JS are also able to perform better in COD tests. Actually, COD speed may be defined as a highly-complex physical quality, which directly depends on numerous motor skills, such as proper technique, straight sprinting speed, reactive strength, concentric strength and power, ability to decelerate and accelerate fast, and left-right muscle imbalance (among other capacities)[37,39]. As a consequence of this complexity, we expected that the distinct levels of performance in these “isolated strength-power exercises” (i.e., HS or JS) would be not able to differentiate the individual performances in COD tests. Again, it is probable that the higher amounts of accelerative forces applied throughout the entire concentric portion of the JS may, to some extent, explain this very interesting neuromechanical phenomenon. This finding may have important implications in sport training.

From the present study, it was not possible to determine the physiological mechanisms explaining the association between JS and jump, sprint, and COD performances in rugby sevens players. However, it can be speculated that muscle architecture, as determined by the size and relative arrangement of fiber fascicles, is significantly correlated with both the ability to perform well in the aforementioned tests (especially in the sprint test [40,41]) and in the JS assessments. Further, it is equally plausible that the rate of force development of athletes with higher performances in loaded and unloaded jumps and in sprint tests is higher than in athletes with lower performances, since all these variables are possibly related to the proportion of fast twitch fibers [42] and neural drive [43,44] (e.g., initial firing rate).

## Conclusions

In summary, JS was shown to be more connected to sprinting, COD speed, and jumping abilities than HS in elite rugby sevens players. Notably, the slight but meaningful mechanical differences between the JS and HS, related to the full application of accelerative forces during the entire concentric portion of the JS exercise and the presence of deceleration during the final stages of the concentric portion of the HS exercise, determine their respective abilities in relation to functional field-based performances. Therefore, JS is suggested to better discriminate between players with higher and lower sprint, and COD and jumping abilities than HS, and possibly training using JS would be more effective than training using HS for improving performance in rugby sevens players. Because of the well-established strong relationship between JS MPP and specific performance in different sports [16,17,19,45,46] and the effectiveness of JS training in improving jumping and sprinting capacities [13,23,35,36], this exercise has been incorporated in assessments and training routines by elite athletes. To our knowledge, this is the first study to show that JS relates more closely to functional task performances than HS using the median split technique. By dividing a group of athletes around the median value, this simple and intuitive method can be used by technical staff to 1) classify their athletes as a group and track prospective changes caused by training/detraining (using the median), 2) identify strengths and weaknesses of individual players (using the individual values in relation to the median) and, 3) help in choosing the most appropriate exercises (i.e., those with a closer relation/connection with specific functional performances) to be implemented in the training routines (e.g., loaded jump squats). Although the final item needs to be confirmed by longitudinal observations, median-split analysis can be used in sport science to raise evidence favoring selected strength-power exercises.

## References

[1] Loturco I, Winckler C, Kobal R, Cal Abad CC, Kitamura K, Verissimo AW, et al Performance changes and relationship between vertical jump measures and actual sprint performance in elite sprinters with visual impairment throughout a Parapan American games training season. Front Physiol. 2015;6: 323 10.3389/fphys.2015.00323 26594181 PMC4635212

[2] Loturco I, Ugrinowitsch C, Roschel H, Lopes Mellinger A, Gomes F, Tricoli V, et al Distinct temporal organizations of the strength- and power-training loads produce similar performance improvements. J Strength Cond Res. 2013;27: 188–194. 10.1519/JSC.0b013e3182503807 22362090

[3] Loturco I, Ugrinowitsch C, Roschel H, Tricoli V, Gonzalez-Badillo JJ. Training at the optimum power zone produces similar performance improvements to traditional strength training. J Sports Sci Med. 2013;12: 109–115. 24149733 PMC3761767

[4] Newton RU, Kraemer WJ. Developing explosive muscular power: Implications for a mixed methods training strategy. Strength Cond J. 1994;16: 20–31.

[5] Cormie P, McGuigan MR, Newton RU. Developing maximal neuromuscular power: part 2—training considerations for improving maximal power production. Sports Med. 2011;41: 125–146. 10.2165/11538500-000000000-00000 21244105

[6] McBride JM, Nimphius S, Erickson TM. The acute effects of heavy-load squats and loaded countermovement jumps on sprint performance. J Strength Cond Res. 2005;19: 893–897. 10.1519/R-16304.1 16287357

[7] Chelly MS, Fathloun M, Cherif N, Ben Amar M, Tabka Z, Van Praagh E. Effects of a back squat training program on leg power, jump, and sprint performances in junior soccer players. J Strength Cond Res. 2009;23: 2241–2249. 10.1519/JSC.0b013e3181b86c40 19826302

[8] Argus CK, Gill ND, Keogh JW, McGuigan MR, Hopkins WG. Effects of two contrast training programs on jump performance in rugby union players during a competition phase. Int J Sports Physiol Perform. 2012;7: 68–75. 22461463 10.1123/ijspp.7.1.68

[9] Barnes C, Archer DT, Hogg B, Bush M, Bradley PS. The evolution of physical and technical performance parameters in the English Premier League. Int J Sports Med. 2014;35: 1095–1100. 10.1055/s-0034-1375695 25009969

[10] Bloomfield J, Polman R, O'Donoghue P. Physical demands of different positions in FA Premier League soccer. J Sports Sci Med. 2007;6: 63–70. 24149226 PMC3778701

[11] Faude O, Koch T, Meyer T. Straight sprinting is the most frequent action in goal situations in professional football. J Sports Sci. 2012;30: 625–631. 10.1080/02640414.2012.665940 22394328

[12] Nibali ML, Chapman DW, Robergs RA, Drinkwater EJ. A rationale for assessing the lower-body power profile in team sport athletes. J Strength Cond Res. 2013;27: 388–397. 10.1519/JSC.0b013e3182576feb 22505130

[13] Loturco I, Pereira LA, Kobal R, Zanetti V, Gil S, Kitamura K, et al Half-squat or jump squat training under optimum power load conditions to counteract power and speed decrements in Brazilian elite soccer players during the preseason. J Sports Sci. 2015;33: 1283–1292. 10.1080/02640414.2015.1022574 25772972

[14] Hori N, Newton RU, Kawamori N, McGuigan MR, Andrews WA, Chapman DW, et al Comparison of weighted jump squat training with and without eccentric braking. J Strength Cond Res. 2008;22: 54–65. 10.1519/JSC.0b013e31815ef052 18296956

[15] Nimphius S, McGuigan MR, Newton RU. Relationship between strength, power, speed, and change of direction performance of female softball players. J Strength Cond Res. 2010;24: 885–895. 10.1519/JSC.0b013e3181d4d41d 20300038

[16] Loturco I, D'Angelo RA, Fernandes V, Gil S, Kobal R, Abad CCC, et al Relationship between sprint ability and loaded/unloaded jump tests in elite sprinters. J Strength Cond Res. 2015;29: 758–764. 10.1519/JSC.0000000000000660 25162648

[17] Loturco I, Pereira LA, Cal Abad CC, D'Angelo RA, Fernandes V, Kitamura K, et al Vertical and horizontal jump tests are strongly associated with competitive performance in 100-m dash events. J Strength Cond Res. 2015;29: 1966–1971. 10.1519/JSC.0000000000000849 25627643

[18] Wisloff U, Castagna C, Helgerud J, Jones R, Hoff J. Strong correlation of maximal squat strength with sprint performance and vertical jump height in elite soccer players. Br J Sports Med. 2004;38: 285–288. 10.1136/bjsm.2002.002071 15155427 PMC1724821

[19] Loturco I, Barbosa AC, Nocentini RK, Pereira LA, Kobal R, Kitamura K, et al A correlational analysis of tethered swimming, swim sprint performance and dry-land power assessments. Int J Sports Med. 2016;37: 211–218. 10.1055/s-0035-1559694 26669251

[20] Stone MH, Stone ME, Sands WA, Pierce KC, Newton RU, Haff GG, et al Maximum strength and strength training: a relationship to endurance? Strength Cond J. 2006;28: 44–53.

[21] Rampinini E, Bishop D, Marcora SM, Ferrari Bravo D, Sassi R, Impellizzeri FM. Validity of simple field tests as indicators of match-related physical performance in top-level professional soccer players. Int J Sports Med. 2007;28: 228–235. 10.1055/s-2006-924340 17024621

[22] Iacobucci D, Posavac SS, Kardes FR, Schneider M, Popovich DL. The median split: robust, refined, and revived. J Consumer Psych Forthcoming. 2015;25: 690–704.

[23] Loturco I, Nakamura FY, Kobal R, Gil S, Cal Abad CC, Cuniyochi R, et al Training for power and speed: effects of increasing or decreasing jump squat velocity in elite young soccer players. J Strength Cond Res. 2015;29: 2771–2779. 10.1519/JSC.0000000000000951 25807028

[24] Loturco I, Nakamura FY, Tricoli V, Kobal R, Abad CC, Kitamura K, et al Determining the optimum power load in jump squats using the mean propulsive velocity. PLoS One. 2015;10: e0140102 10.1371/journal.pone.0140102 26444293 PMC4596801

[25] Sanchez-Medina L, Perez CE, Gonzalez-Badillo JJ. Importance of the propulsive phase in strength assessment. Int J Sports Med. 2010;31: 123–129. 10.1055/s-0029-1242815 20222005

[26] Little T, Williams AG. Specificity of acceleration, maximum speed, and agility in professional soccer players. J Strength Cond Res. 2005;19: 76–78. 10.1519/14253.1 15705049

[27] Batterham AM, Hopkins WG. Making meaningful inferences about magnitudes. Int J Sports Physiol Perform. 2006;1: 50–57. 19114737

[28] Cohen J. Statistical power analysis for the behavioral sciences. Hillsdale (NJ): Lawrence Erlbaum Associates; 1988.

[29] Rhea MR. Determining the magnitude of treatment effects in strength training research through the use of the effect size. J Strength Cond Res. 2004;18: 918–920. 10.1519/14403.1 15574101

[30] Cormie P, McCaulley GO, Triplett NT, McBride JM. Optimal loading for maximal power output during lower-body resistance exercises. Med Sci Sports Exerc. 2007;39: 340–349. 10.1249/01.mss.0000246993.71599.bf 17277599

[31] Newton RU, Kraemer WJ, Hakkinen K, Humphries B, Murphy AJ. Kinematics, kinetics, and muscle activation during explosive upper body movements. J Appl Biomech. 1996;12: 31–43. 10.1007/s004210050169 9134365

[32] Cronin JB, Hansen KT. Strength and power predictors of sports speed. J Strength Cond Res. 2005;19: 349–357. 10.1519/14323.1 15903374

[33] Baker D, Nance S. The relation between strength and power in professional rugby league players. J Strength Cond Res. 1999;13: 224–229.

[34] Costill DL, Miller SJ, Myers WC, Kehoe FM, Hoffman WM. Relationship among selected tests of explosive leg strength and power. Res Quart Amer Ass Health Physical Ed Rec. 1968;39: 785–787.

[35] McBride JM, Triplett-McBride T, Davie A, Newton RU. The effect of heavy- vs. light-load jump squats on the development of strength, power, and speed. J Strength Cond Res. 2002;16: 75–82. 11834109

[36] Loturco I, Pereira LA, Kobal R, Maldonado T, Piazzi AF, Bottino A, et al Improving sprint performance in soccer: effectiveness of jump squat and Olympic push press exercises. PLoS One. 2016;11: e0153958 10.1371/journal.pone.0153958 27100085 PMC4839661

[37] Sheppard JM, Young WB. Agility literature review: classifications, training and testing. J Sports Sci. 2006;24: 919–932. 10.1080/02640410500457109 16882626

[38] Young W, Farrow D. A review of agility: practical applications for strength and conditioning. Strength Cond J. 2006;28: 24–29.

[39] Hewit JK, Cronin JB, Hume PA. Understanding change of direction performance: a technical analysis of a 180° aerial catch and turn task. Int J Sports Sci Coaching. 2012;7: 503–514.

[40] Kumagai K, Abe T, Brechue WF, Ryushi T, Takano S, Mizuno M. Sprint performance is related to muscle fascicle length in male 100-m sprinters. J Appl Physiol (1985). 2000;88: 811–816. 10710372 10.1152/jappl.2000.88.3.811

[41] Nimphius S, McGuigan MR, Newton RU. Changes in muscle architecture and performance during a competitive season in female softball players. J Strength Cond Res. 2012;26: 2655–2666. 10.1519/JSC.0b013e318269f81e 22847524

[42] Viitasalo JT, Komi PV. Force-time characteristics and fiber composition in human leg extensor muscles. Eur J Appl Physiol Occup Physiol. 1978;40: 7–15. 569577 10.1007/BF00420984

[43] Aagaard P. Training-induced changes in neural function. Exerc Sport Sci Rev. 2003;31: 61–67. 12715968 10.1097/00003677-200304000-00002

[44] Aagaard P, Simonsen EB, Andersen JL, Magnusson P, Dyhre-Poulsen P. Increased rate of force development and neural drive of human skeletal muscle following resistance training. J Appl Physiol. 2002;93: 1318–1326. 10.1152/japplphysiol.00283.2002 12235031

[45] Loturco I, Kobal R, Maldonado T, Piazzi AF, Bottino A, Kitamura K, et al Jump squat is more related to sprinting and jumping abilities than Olympic push press. Int J Sports Med. 2015;In Press. 10.1055/s-0035-1565201 26667925

[46] Loturco I, Nakamura FY, Artioli GG, Kobal R, Kitamura K, Cal Abad CC, et al Strength and power qualities are highly associated with punching impact in elite amateur boxers. J Strength Cond Res. 2016;30: 109–116. 10.1519/JSC.0000000000001075 26110348

